# A new genus of nemonychid weevil from Burmese amber (Coleoptera, Curculionoidea)

**DOI:** 10.3897/zookeys.405.6475

**Published:** 2014-04-28

**Authors:** Steven R. Davis, Michael S. Engel

**Affiliations:** 1 Division of Entomology, Natural History Museum, and Department of Ecology & Evolutionary Biology, 1501 Crestline Drive – Suite 140, University of Kansas, Lawrence, Kansas 66045, USA

**Keywords:** Coleoptera, amber, Cretaceous, weevils, Nemonychidae, Mesozoic, taxonomy

## Abstract

The first fossil nemonychid (Nemonychidae) in Burmese amber, belonging to the subfamily Rhinorhynchinae, is described and figured as *Burmonyx zigrasi* Davis & Engel, **gen. n.** and **sp. n.** While this specimen also comprises the first definitive record of the subfamily in the Asian continent, other compression fossils exist at least from the Yixian Formation of China and the Karatau site of Kazakhstan which may also deserve placement within this group. Although several important areas of the body are obscured by the shape and fragmented condition of the amber piece, a sufficient number of features are visible to consider adequate placement within Rhinorhynchinae, including the fairly strongly punctate elytral striae and appendiculate, nearly bifid pretarsal claws.

## Introduction

The origin and evolution of weevils (Curculionoidea) remains one of the more interesting and challenging areas of systematic research among the Coleoptera (Grimaldi and Engel 2005). The distinctive weevil rostrum has been implicated as a key innovation intimately tied to their considerable success in terms of species and ecological diversity (Anderson 1995). Although much remains to be illuminated regarding the larger patterns of weevil phylogeny, one thing is certain: among the extant families, the Nemonychidae are the most basal offshoot of the lineage (Kuschel 1995; Oberprieler et al. 2007). Often familiarly known as pine cone weevils owing to their association with diet of pollen from pines and related gymnosperms, nemonychids primitively retain non-geniculate (orthocerous) antennae, lack pronotal lateral carinae (except apparently in the nemonychid subfamily Eobelinae), and have the abdominal ventrites separate (rather than partially fused or entirely connate). Given this critical phylogenetic position, it is understandable why nemonychids play a large role in narratives regarding the early order of weevils (e.g., Legalov 2010b, 2012; Oberprieler et al. 2007).

**Figures 1–6.**
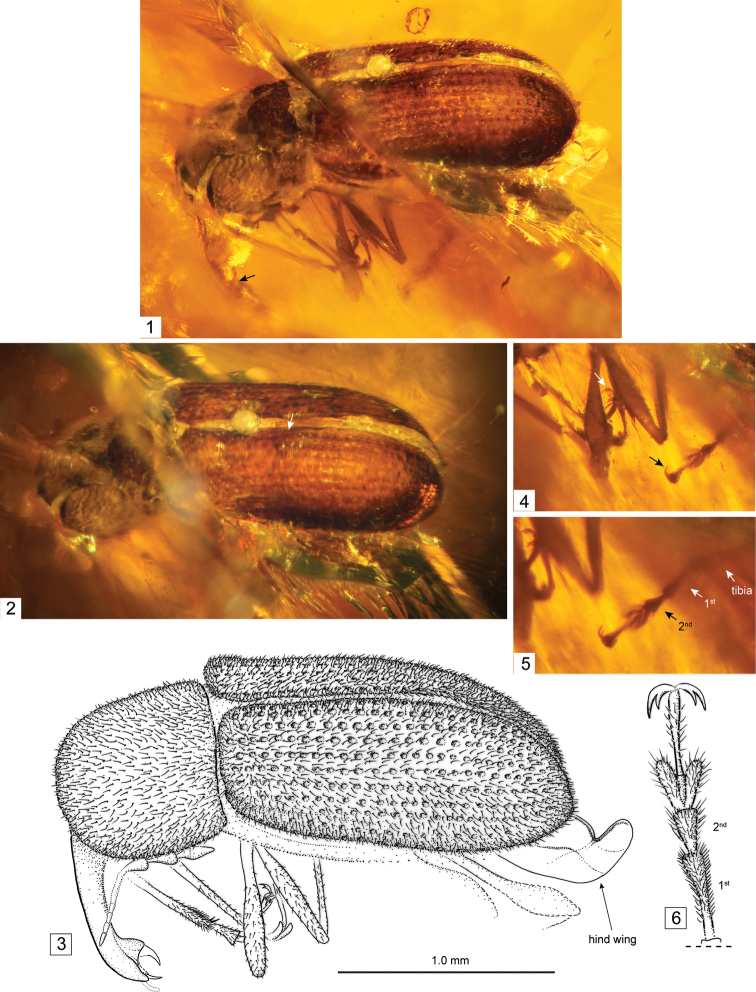
Photomicrographs (**1, 2, 4, 5**) and line drawings (**3, 6**) of holotype of *Burmonyx zigrasi* Davis and Engel, sp. n. (JZC-Bu228). **1** Dorso-lateral view of specimen inclusion, arrow pointing to antennal scape **2** Slightly more dorsal view of specimen than in figure 1, arrow pointing to scutellary striole **3** Line drawing of specimen (scale bar only applies to this figure) **4** Legs, arrows pointing to appendiculate, nearly bifid pretarsal claws **5** Enlargement of legs, arrows pointing to metatibia and metatarsomeres **6** Illustration of metatarsus.

Thorough accounts of the Mesozoic fossil record of Nemonychidae (and of the superfamily Curculionoidea) have been provided and reiterated several times (e.g., Kuschel 1983; Kuschel and Leschen 2011; Oberprieler and Oberprieler 2012; Riedel 2010), therefore it will not be repeated here. Extant members of the subfamily Rhinorhynchinae are distributed in the southwest Nearctic, Neotropical, and Australian regions (Kuschel and Leschen 2011). While there appear to be fossil representatives of this subfamily in various deposits in Asia (e.g., Karatau, Yixian), Asian fossil taxa have yet to be ascribed to this lineage. The description of the new genus and species herein represents the first nemonychid found in Burmese amber (Figs 1, 2), as well as the first record of the subfamily Rhinorhynchinae in Asia.

## Material and methods

The amber piece containing the holotype was excavated from the strata in the northern state of Kachin in Myanmar as part of regular and ongoing mining operations and is from the collection of Mr. James S. Zigras, available for study through the American Museum of Natural History (AMNH), New York. The origin, age, and fauna of Burmese amber have been reviewed by Grimaldi et al. (2002), Ross et al. (2010), and Shi et al. (2012), the latter of which arrived at an age of approximately 99 Ma (providing a range close to the Aptian-Cenomanian boundary). Due to the presence of several other insect inclusions and the heavily fragmented state of the amber piece, further preparation beyond some initial polishing was not possible, leaving some aspects of the specimen unobservable or obscured. Due to the round surface of the piece, glycerin was applied to a small coverslip and placed on the area directly above the inclusion in order to acquire more satisfactory viewing and photography. Photomicrographs were obtained by combining a z-stack of around 25 images using the computer software CombineZ. Illustrations were made through the aid of a drawing tube attached to an Olympus SZX9 stereomicroscope. The general familial classification of Curculionoidea follows that of Oberprieler et al. (2007).

## Systematic paleontology

### Family Nemonychidae Bedel
Subfamily Rhinorhynchinae Voss

#### Burmonyx

Davis & Engel gen. n.

http://zoobank.org/C7493C8C-E242-4EC9-BD39-9BC4C4C579F9

http://species-id.net/wiki/Burmonyx

##### Type species.

*Burmonyx zigrasi* Davis & Engel, sp. n. Gender feminine.

##### Diagnosis.

The new genus appears similar to several genera in Rhinorhynchinae, particularly to those of Rhinorhynchini, due to the fairly long, narrow rostrum (Figs 1, 3) and moderately wide pronotum. It appears to be differentiated based mainly on its elongate scutellary striole. The striole in extant Nemonychidae always is fairly short and not extending more than 0.25 × the length of the elytra from the base. This genus, however, possesses a striole that extends approximately to the middle of the elytron. The relatively large strial punctures on the elytra may also be a defining feature of this genus, as the punctures typically are quite small in other members of Rhinorhynchinae.

##### Etymology.

The genus name is a combination of the specimen’s collection locality, Burma (former name of Myanmar), and the Greek *nyx*, meaning “night”.

#### Burmonyx zigrasi

Davis & Engel sp. n.

http://zoobank.org/15B9C6E0-166E-4D25-A142-5B8E12517468

http://species-id.net/wiki/Burmonyx_zigrasi

Figs 1 –7

##### Holotype.

JZC-Bu228, Myanmar: Kachin; Cretaceous: Early Cenomanian; in the private collection of Mr. James S. Zigras, available for study through the Division of Invertebrate Zoology, American Museum of Natural History (AMNH), New York, USA. Material was obtained from ongoing excavations in mines in the Hukawng Valley, northern Myanmar (Grimaldi et al. 2002). The raw material was brought back to Myitkyina where initial polishing and sorting was undertaken prior to final preparation of pieces in the AMNH.

##### Diagnosis.

As for the genus (*vide supra*).

##### Description.

**Figures 7–13.**
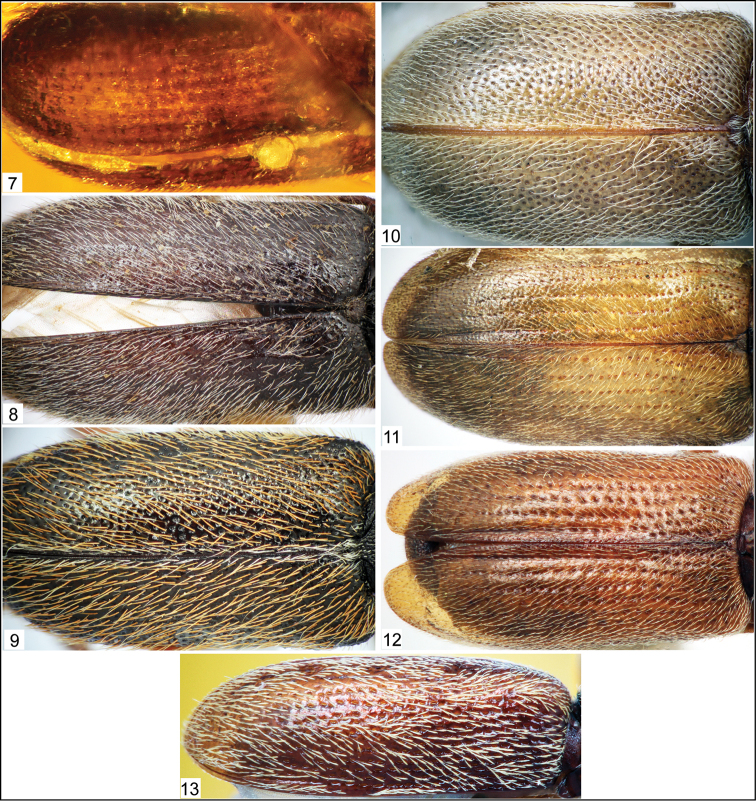
Photomicrographs of nemonychid elytra (dorsal aspect). **7** *Burmonyx zigrasi* Davis and Engel, sp. n. **8** *Nemonyx lepturoides* (Fabricius, 1801) **9** *Cimberis elongata* (LeConte, 1876) **10** *Doydirhynchus austriacus* (Olivier, 1807) **11** *Basiliorhinus araucariae* Kuschel, 1994 **12** *Nannomacer germaini* (Voss, 1952) **13** *Rhinorhynchus rufulus* (Broun, 1880).

Total body length (excluding rostrum): ca. 2.2 mm; maximal width (along middle of elytra): ca. 0.7 mm; elytral length: ca. 1.3 mm. Integument appearing light to dark brown (Figs 1, 2, 7). Scales absent, but dense covering of setae along at least pronotum and elytra (lateral and ventral surfaces not clearly visible). Head and compound eyes not clearly visible (slightly pushed into and obscured by prothorax). Rostrum approximately as long as pronotum along middle (exact length ratio unclear due to obscured head), fairly slender, abruptly widening apically (Fig. 3). Mandibles large, falciform. Antennae orthocerous, inserted dorso-laterally at apical 1/4; clubs composed of 3 loose articles. Pronotum seemingly as wide or nearly as wide as elytral humeri; not constricted anteriorly at collar, slightly rugulose, bearing small, dense, shallow punctures. Mesoscutellum not visible. Elytra with ten shallowly punctate striae (Figs 2, 3, 7); scutellary striole present, extending approximately to mid-length of elytra; interstices lacking punctures; elytral shoulders rounded. Abdomen with pygidium (tergite VII) concealed. Legs approximately equal in length, slender; femora slender; trochanters small, triangular; tibial spur formula 2-2-2; tarsomere 1 rather narrow, elongate (Fig. 6), approximately 2 × as long as tarsomere 2, 2 with rounded apico-lateral margins; 3 strongly bilobed (Figs 5, 6), lobes narrow; 4 short, slightly longer than 0.5 × length of tarsomere 3; 5 slender, approximately 2 × as long as tarsomere 2; pretarsal claws (ungues) divaricate strongly appendiculate, nearly bifid (Figs 4, 6).

##### Etymology.

The specific epithet is dedicated to the collector, Mr. James S. Zigras, who permitted study of the material and has generously supported amber research.

## Discussion

Presence of scutellary strioles excludes a placement of *Burmonyx* in Caridae, Brentidae, and Curculionidae. However, as the dorsal area encompassing the elytral suture is somewhat difficult to observe, if the presence of strioles represents a misinterpretation of complete striae, a superficial resemblance to the group Mesophyletinae Poinar (2006) may be assumed primarily in the shape of the tarsomeres and divaricate bifid pretarsal claws. Beyond these similarities, *Burmonyx* possesses orthocerous antennae (in which the scape is short and similar in length to subsequent antennomeres) and tibiae with smooth dorsal margins, sufficient information to exclude such a placement. The absence of a distinct pygidium, incompletely covered by the elytra and typically extending ventrad, and the conical second tarsomeres that do not envelope the base of the third, excludes it from Anthribidae and Attelabidae.

In Nemonychidae, as members of Nemonychinae do not possess distinctly punctate striae (Fig. 8), those of Cimberidinae lack striae (Figs 9–10), and both groups lack elytral strioles, *Burmonyx* does not belong within those subfamilies and a possible placing within Rhinorhynchinae or Eobelinae remains. Eobelinae are a difficult assemblage of taxa to comprehend in the least, and it is yet rather unclear how these different groups relate to the extant nemonychid fauna. It is clear that many of the taxa, which appear to have simple, divaricate pretarsal claws and a rostrum emerging from the ventral part of the head capsule, also bear distinctly punctate elytral striae, and may also bear scutellary strioles (Table 1; Figs 20–29). It also is interesting to note that there are some undescribed taxa from the Jurassic deposits of Karatau (Kazakhstan) and Daohugou (Inner Mongolia, China) that have a dense scattering of elytral punctures and lack elytral striae (appearing to also lack scutellary strioles), similar to extant Cimberidinae. Nonetheless, *Burmonyx* can be excluded from Eobelinae based on its appendiculate, almost bifid pretarsal claws.

**Table 1.** Exemplar taxa sampled from the four recognized subfamilies of Nemonychidae (Alonso-Zarazaga and Lyal 1999; Kuschel and Leschen 2011), summarizing the layout of punctures on the elytra and indicating the presence of a scutellary striole.

Subfamily: Tribe	Species	Elytron puncture type
Nemonychinae	*Nemonyx lepturoides* (Fabricius, 1801)	Punctures scattered, striae present, faint; striole absent
Cimberidinae: Cimberidini	*Cimberis elongata* (LeConte, 1876)	Punctures scattered, striae absent; striole absent
Doydirhynchini	*Doydirhynchus austriacus* (Olivier, 1807)	Punctures scattered, striae absent; striole absent
Rhinorhynchinae: Rhinorhynchini	*Basiliorhinus araucariae* Kuschel, 1994	Punctures aligned into striae; striole present
	*Nannomacer germaini* (Voss, 1952)	Punctures aligned into striae; striole present
	*Rhinorhynchus rufulus* (Broun, 1880)	Punctures aligned into striae; striole present
	*Rhynchitomacer flavus* Voss, 1937	Punctures aligned into striae; striole present
Rhinorhynchini?	*Burmonyx zigrasi* Davis & Engel, **sp. n.**	Punctures aligned into striae; striole present
Mecomacerini	*Aragomacer leai* Kuschel, 1994	Punctures aligned into striae; striole present
	*Mecomacer scambus* Kuschel, 1954	Punctures aligned into striae; striole present
	*Brarus mystes* Kuschel, 1997	Punctures aligned into striae; striole present
	*Rhynchitomacerinus kuscheli* (Voss, 1952)	Punctures aligned into striae; striole present
	*Rhynchitoplesius eximius* (Voss, 1952)	Punctures aligned into striae; striole present
Eobelinae: Eobelini	*Eobelus longipes* Arnol’di, 1977	Punctures aligned into striae; striole present
	*Archaeorrhynchus paradoxopus* Arnol’di, 1977	Punctures aligned into striae; striole present
Oxycorynoidini	*Oxycorynoides similis* Arnol’di, 1977	Punctures aligned into striae; striole present
Brenthorrhinoidini	*Brenthorrhinoides mandibulatus* Gratshev & Zherikhin, 1996	Punctures aligned into striae; striole present
Distenorrhinini	*Distenorrhinus* spp.	Punctures aligned into striae; striole present

**Figures 14–19.**
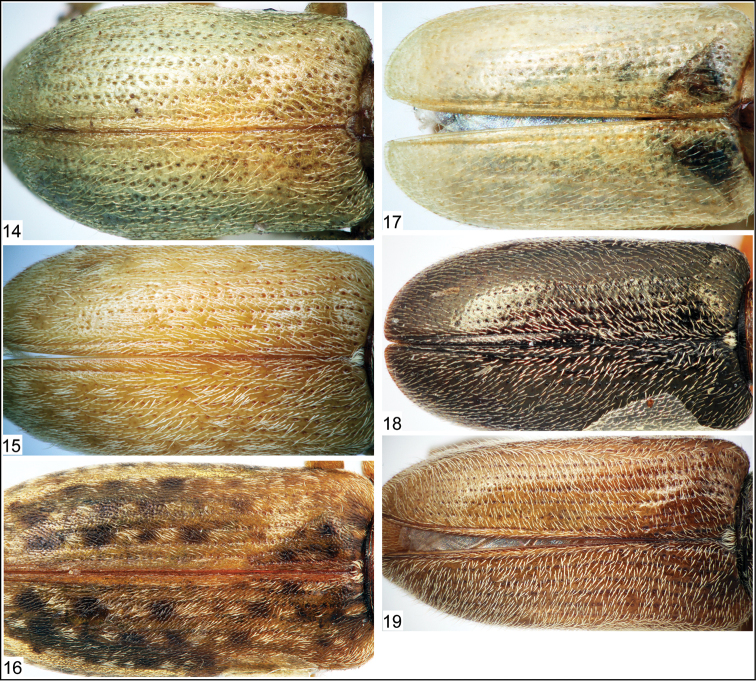
Photomicrographs of nemonychid elytra (dorsal aspect). **14** *Rhynchitomacer flavus* Voss, 1937 **15** *Aragomacer leai* Kuschel, 1994 **16** *Mecomacer scambus* Kuschel, 1954 **17** *Brarus mystes* Kuschel, 1997 **18** *Rhynchitomacerinus kuscheli* (Voss, 1952) **19** *Rhynchitoplesius eximius* (Voss, 1952).

Kuschel (1995) and Kuschel and Leschen (2011) listed several synapomorphies for Rhinorhynchinae, of which only a few are visible in *Burmonyx*. In this new genus, the elytral punctures are distinctly aligned into striae and the pretarsal claws are appendiculate, both characters specifying its placement to Rhinorhynchinae. Although the visible features of this specimen may also superficially appear to allow placement within Nemonychinae, the elytral striae in the latter subfamily are much less defined and the elytra contain a scattering of small punctures. In Rhinorhynchinae, all species have well-defined punctate elytral striae (Figs 11–19). According to its rather elongate, narrow rostrum, the fairly large strial punctures of the elytra, as well as the somewhat wide pronotum in relation to the width of the elytra, *Burmonyx* seems to be more closely related to taxa of Rhinorhynchini; however, the nearly bifid pretarsal claws also bear some likeness to those members of Mecomacerini. As the head also appears to be pushed into the pronotum in this specimen or at least concealed in dorso-lateral view by a small portion of the pronotum, it is possible that the compound eyes are not prominent in *Burmonyx*, though this statement is not verifiable due to the obscured head region. Alas, given that much of the head, a large portion of the rostrum and most detail of the mouthparts are obscured in this specimen (not to mention lack of visibility of any internal features), an informed tribal placement within Rhinorhynchinae seems rather implausible.

**Figures 20–23.**
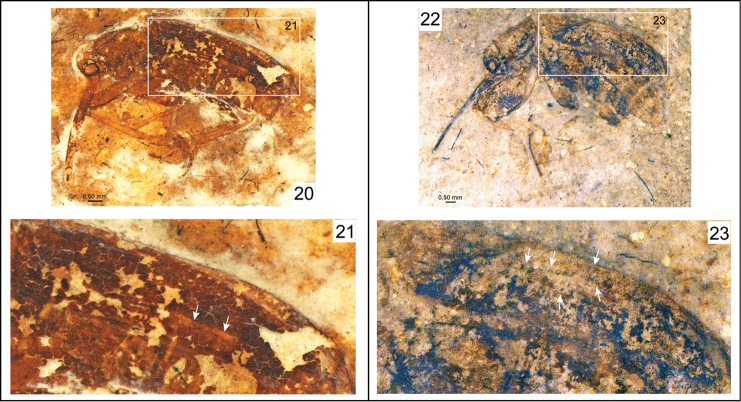
Photomicrographs of fossil nemonychid taxa and their elytra. **20** *Eobelus longipes* Arnol’di, 1977 (holotype), PIN 2452-275 **21** Enlargement of outlined elytron in Figure 20 **22** *Archaeorrhynchus paradoxopus* Arnol’di, 1977 (holotype), PIN 2335-42 **23** Enlargement of outlined elytron in Figure 22.

**Figures 24–29.**
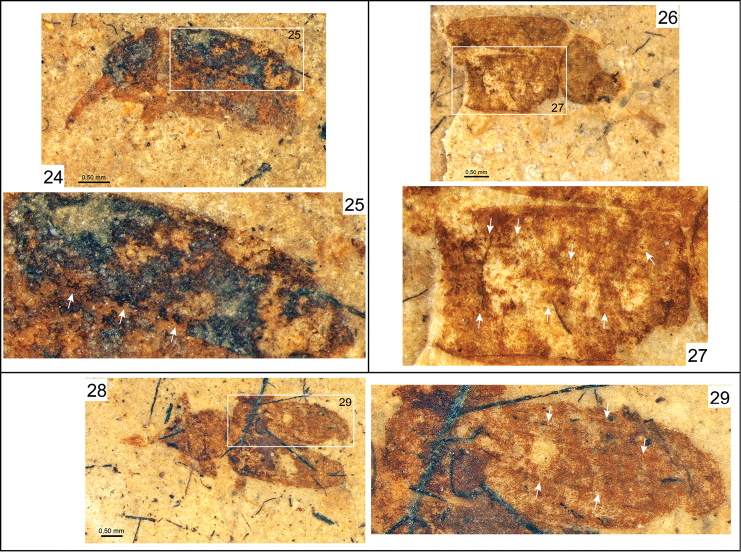
Photomicrographs of fossil nemonychid taxa and their elytra. **24** *Oxycorynoides similis* Arnol’di, 1977 (holotype), PIN 2554-713 **25** Enlargement of outlined elytron in Figure 24 **26** *Brenthorrhinoides mandibulatus* Gratshev and Zherikhin, 1996 (holotype), PIN 2239-1508 **27** Enlargement of outlined elytron in Figure 26 **28** *Distenorrhinus* sp., PIN 2239-1547 **29** Enlargement of outlined elytron in Figure 28.

The only nemonychids described from amber inclusions thus far include *Kuschelomacer kerneggeri* Riedel (2010), in Baltic amber, and *Libanorhinus succinus* Kuschel and Poinar (1993), in Lebanese amber. Although *Burmonyx* accounts for the first definitive fossil recorded in the subfamily Rhinorhynchinae (and possibly in Rhinorhynchini) from the Asian continent (certainly the first record in Burmese amber), Kuschel and Leschen (2011) transferred *Cratomacer* Zherikhin and Gratshev (2004) from its original placement in Rhinorhynchini (Rhinorhynchinae) to Mecomacerini, which includes two fossil species, *Cratomacer immersus* Zherikhin and Gratshev (2004) and *Cratomacer ephippiger* Zherikhin and Gratshev (2004). Fossils of Cimberidinae are now known from the Yixian Formation of China (Davis et al. 2013), and the Karatau site also appears to contain members of this subfamily (e.g., *Chinocimberis* Legalov 2009) and Rhinorhynchinae (personal observation), in addition to the well-known Eobelinae. This growing list also includes the recently described Late Jurassic compression fossil *Talbragarus averyi* Oberprieler and Oberprieler (2012), which possibly belongs to either Rhinorhynchinae or Eobelinae. While Karatau certainly has yielded an impressive fauna of nemonychid fossils, this diversity remains a complicated assemblage and the taxa have been placed in several disparate groups based on characters of somewhat questionable interpretation and informativeness (e.g., Arnol’di 1977; Gratshev and Legalov 2009; Legalov 2010a, 2010b, 2012). It also appears that the Yixian Formation may contain fossils of Rhinorhynchinae, as represented by *Abrocar* Liu & Ren (2006); Davis et al. 2013). Although the studies thus far have made valorous strides in sorting this particular fossil fauna, more rigorous examinations of characters are required. Just as greater scrutiny is needed for more accurate placement of taxa, it is possible that in many cases, the level of preservation necessary for acute taxonomic identification (for example, beyond the subfamilial or tribal level) simply is absent and should be recognized, therefore further attempts to classify such taxa must be abandoned.

## Supplementary Material

XML Treatment for Burmonyx

XML Treatment for Burmonyx zigrasi
